# High frequency of alpha7-HPV in Colombian Caribbean coast women: cervical cancer screening analysis

**DOI:** 10.1186/s12879-024-09410-0

**Published:** 2024-05-29

**Authors:** Heiser Arteaga-Pautt, O. Elias Bru-Cordero, Dina Ricardo-Caldera, Lyda Espitia-Pérez, Paula Avilés-Vergara, Catalina Tovar-Acero, Lorena Castaño-Caraballo, Sandra Janeth Perdomo-Lara, Helvey Ramón Zetién-Arteaga, Valentina Behaine-Bravo, Sara Cecilia Soto-De León

**Affiliations:** 1 Grupo de Investigación Enfermedades Tropicales y Resistencia Bacteriana, Universidad del Sinú E.B.Z, Montería, 230001 Colombia; 2 Universidad Nacional de Colombia. Dirección Académica, Km 9 via Valledupar - La Paz, sede de La Paz, La Paz, Cesar, Colombia; 3 Grupo de Investigación Biomédicas y Biología Molecular, Universidad del Sinú E.B.Z, Montería, 230001 Colombia; 4 Liga Cordobesa Contra el Cáncer, Montería-Córdoba, Colombia; 5 Unit of Basic Oral Investigations-UIBO, School of Dentistry, Universidad El Bosque, Bogotá, Colombia; 6 Grupo de investigación Oncogen, Upqua SAS, Bogotá, Colombia; 7 Researcher Biomedical and Molecular Biology Laboratory, Faculty of Basic Sciences of Health, Universidad del Sinú, Montería-Córdoba, Colombia

**Keywords:** High-risk human papillomavirus (HR-HPV), Cervical cancer (CC), Pap smear test, Population, Colombia

## Abstract

**Background:**

Cervical cancer (CC) is a significant global public health concern, particularly in developing countries such as Colombia. The main risk factor involves high-risk HPV types (HR-HPV) infection, coupled with population-specific variables. The Caribbean region in Colombia lacks research on HR-HPV-type frequencies. Therefore, this study aims to establish the prevalence of type-specific HR-HPV and its association with sociodemographic factors among women undergoing cervical cytology screening.

**Methods:**

A cross-sectional study involving voluntary women who provided informed consent and completed a questionnaire capturing sociodemographic, clinical, and sexual behavior information was conducted. All participants underwent cervical cytology and molecular analysis. Generic HPV detection employed three simultaneous PCRs (GP5+/6+, MY09/11, and PU1R/2 M), and positive samples were genotyped using the Optiplex HPV Genotyping kit. The analysis encompassed the 12 types of high-risk HPV (HR-HPV-16,-18,-31,-33,-35,-39,-45,-51,-52,-56,-58, and − 59). Frequencies were reported based on geographic subregions within the Córdoba department, and disparities were made between single and multiple infections. Sociodemographic and clinical variables were subjected to ordinal logistic regression, with statistical significance at a p-value < 0.05. The statistical analyses utilized STATA 14® and R-Core Team-software.

**Results:**

We included 450 women, mean age 40 (SD$\pm$11.44). PCR analysis revealed 43% HPV-positive (*n*=192). GP5+/6+ detected the most positives at 26% (*n*=119), followed by PU1R/2 M at 22% (*n* = 100) and MY09/11 at 15% (*n*=69). Multiple infections occurred in 87.3% (*n*=142), primarily 2 to 4 types (47.37%, *n*=90). Dominant types were HPV-18 (15.6%, *n*=61), HPV-16 (14.9%, *n*=58), HPV-31 (13.0%, *n* = 51), and HPV-45 (11.5%, *n*=45). Logistic regression identified age above 60 as a risk for concurrent multiple types (OR=6.10; 95% CI 1.18–31.63). Menopause was protective (OR=0.31; 95% CI 0.11–0.89).

**Conclusions:**

Our study reveals a notable prevalence of multiple (2–4) high-risk HPV infections among adult women engaged in CC detection initiatives. Predominantly, α7 species constitute the prevalent HR-viral types, with the Medio Sinú subregion showing elevated prevalence. Menopausal status confers protection against diverse HR-HPV infections. Nevertheless, advancing age, particularly beyond 60 years, is linked to an increased susceptibility to simultaneous infections by multiple HPV-types.

## Background

Human Papillomavirus (HPV) is the causal agent of cervical cancer (CC), ranking fourth among the most common types of cancer affecting women. It constitutes a significant public health issue, with an estimated 604,000 new cases and 342,000 deaths worldwide in 2020 [1].

The primary risk factor associated with CC development is persistent HPV infection, representing one of the most common sexually transmitted infections (STIs). HPV encompasses more than 200 viral types, where those known as high-risk (HR-HPV) are the leading cause of neoplastic lesion development. HPV-16 and − 18 are the most significant, followed by HPV-31, 33, 35, 39, 45, 51, 52, 56, 58, 59, and 68 [2, 3]; however, type-specific frequencies vary according to the population type and geographical region analyzed [4]. Additionally, cancer development is multifactorial, influenced by various risk factors associated with sexual behavior and genetic, cultural, and social characteristics of the studied communities [5], and intrinsic characteristics of the viruses present in the infections [6].

Due to the asymptomatic nature of active viral infection in early stages, the presence of HPV is determined when cellular morphological changes occur (cervical abnormalities), for which the Papanicolaou technique (Pap test) is employed in national-level programs for the promotion and prevention of CC [7]. These programs are characterized by adhering to standards such as achieving minimum coverage levels of 80%, ensuring follow-up of abnormal findings with the Pap test (cervical cytology) until confirmatory diagnosis and treatment if necessary, maintaining quality control in all processes, and ultimately monitoring actions in terms of incidence and mortality [8].

It has been demonstrated that identifying HPV empowers women in managing their health. Therefore, including molecular HPV identification in medical care protocols has been instrumental in reducing cervical cancer cases globally [9–11]. Likewise, this type of analysis in cases before cervical lesion development would help decrease the number of colposcopies performed and tailor cervical cancer management guidelines based on the epidemiology and clinical characteristics of the specific geographic region [12].

The clinical importance of infections involving more than one type of HPV has not been fully understood. Some studies suggest that a lesion might be attributed to a single type of HPV [13], while others have shown a high proportion of women with cervical lesions harboring more than one type of HPV [14, 15]. Considering that patients infected with multiple HPV types require ongoing monitoring to prevent the progression of cervical lesions to cancer, detecting the genotype and quantity of HPV could offer critical insights into diagnosing infections and managing and predicting patients’ outcomes in the future.

In Colombia, 30,997 cases of cervical cancer have been reported, showing a continuous upward trend. By 2022, there was a 17% increase compared to 2021. Between January 2, 2021, and January 1, 2022, 2,587 new cases were reported, 71.4% corresponding to invasive cancer, and 92% were staged as II and III. The highest proportion of new cases occurred in women aged 37 to 59. The regions with the highest concentration of these cases were the Caribbean region, with 24.1%, and the Central region, with 21.3% [16].

According to the Colombian National Public Health Surveillance System (SIVIGILA), the department of Córdoba reported 1,675 new cases between 2017 and 2021, averaging 335 cases annually. In Monteria’s municipality, 649 new cases were reported during the same period, averaging 129.8 annually, representing 38.74% of the total cases in Cordoba’s department [16].

For two decades, several independent studies have investigated the prevalence of HPV in the Colombian population [17–20]. However, it’s noteworthy that these studies have yet to address the Colombian Caribbean coastal region specifically. The Colombian Caribbean is characterized by its population heterogeneity and diverse clinical conditions. The region hosts a unique racial and cultural mix, influenced by indigenous, Spanish, and African peoples, with significant migration due to its bordering proximity to countries like Venezuela and Panama, resulting in a substantial population flow. Its climate variations and precipitation patterns also render it an area with eco-epidemiological features and population characteristics distinct from other regions in the country [21]. Historically, this region has faced adverse conditions, experiencing high poverty indicators and complex violence situations that have affected healthcare investment and service conditions [22].

This research aims to determine the frequency of the 12 h-HPV genotypes considered carcinogenic within group 1 A for CC [23], established by the IARC, in women attending cervical cytology screenings at the CLAC. Additionally, it aims to assess their association with clinical and sociodemographic findings in the Colombian subregions of Córdoba, Sucre, and Urabá.

## Methodology

The study employed an analytical cross-sectional observational design. Samples were gathered between March and May 2019 from women attending cervical cytology screenings at the IPS Liga Cordobesa Against Cancer (CLAC). Participants received detailed information about the study, completed a questionnaire detailing their sociodemographic characteristics and sexual behavior, and provided their cervical cytology brush for molecular analyses. This study has received ethical approval from the Investigation and Ethics Review Committee of the Universidad del Sinú. It adheres to the principles described in the Declaration of Helsinki. Adult women who voluntarily gave informed consent participated in the research.

Exclusion criteria comprised pregnant women, individuals with physical impediments affecting sample collection, those who had undergone hysterectomy, those lacking a cervix, or cases where the sample did not meet the required volume or quality.

### Collection of cervical samples and PCR-based HPV detection

Trained personnel collected cervical cytology samples at the LCCC facilities. These results were subsequently utilized for the population study. The cervical epithelium samples were obtained using a cervical brush and then preserved in glass tubes containing 2.5 ml of 95% ethanol, stored at a temperature of 4 °C.

The DNA extraction was conducted at the Biomedical Research Laboratory of the University of Sinú- Elías Bechara Zainum, using the Quick-DNA Miniprep plus kit for cervical samples. HPV was determined using the Polymerase Chain Reaction (PCR) technique through three independent protocols. The primers GP5+/6 + and MY09/11 were employed to identify the L1 region of the virus, while pU1R/2 M targeted the E6 protein of high-risk types. DNAase-free water (GIBCO) served as the negative control, and previously identified HPV-positive samples by the Molecular Biology Group of the Institute of Immunology of Colombia Foundation (FIDIC) were used as positive controls [17].

### Multiple HPV genotyping using luminex® technology

The DNA extracted from HPV-positive samples was amplified using PCR. Biotinylated primer sets BGP5/BGP6, included in the kit, were employed for this amplification. Additionally, a sample control involved amplifying a fragment of the ß-globin gene.

The products generated by PCR were combined with a microsphere encompassing 21 types of HPV (-6, -11, -16, -18, -26, -31, -33, -35, -39, -44, -45, -51, -52, -53, -56, -58, -59, -66, -68, -70, -73, -82), a representation of ß-globin, and an oligonucleotide probe for hybridization control. ß-globin’s presence was a quality check for genomic DNA in the PCR. After thermal denaturation and hybridization of the target sequences to the probes linked to the microspheres, the biotinylated PCR products adhered to the microspheres were labeled using streptavidin, functioning as a fluorescence reporter with R-Phycoerythrin labeling. Finally, the microspheres were analyzed using a Luminex 100 reader (Luminex Corp).

Using the detection of specific HPV and PCR $\beta$-globin products as a basis, the assessment followed this scheme: The cutoff value for each sequence and HPV was calculated considering the signal from the negative control, with the condition that the standard signal values for hybridization control were above 200. The outcomes in this article are expressed in binary terms (presence or absence).

### Statistical analysis

The study variables were analyzed using descriptive statistical methods, employing measures like mean for central tendency and standard deviation for dispersion. All frequencies and 95% confidence intervals (CI) were presented as percentages. Sociodemographic factors such as age, education level, ethnicity, contraceptive usage, smoking habits, number of pregnancies, age at first sexual intercourse, and count of sexual partners were treated as categorical variables and expressed in terms of frequencies. The subregion variable utilized the department’s geopolitical distribution data [24], organizing samples based on patient origin to highlight respective frequencies.

The results of HR-HPV infections, identified through molecular means, were categorized by the number of simultaneous viral types in each sample. For the association analysis, the HPV infection status served as the dependent variable, categorized into three groups: single infections, multiple infections (2 to 4 types), and various infections (5 or more types) simultaneously. Sociodemographic and clinical variables were treated as independent variables [25]. Using OR and their respective 95% CI, the association strength was evaluated using ordinal logistic regression methods. Statistical procedures were conducted using STATA 14® and R Core Team® [26].

## Results

The study examined a cohort of 450 voluntary women who participated by completing a survey consolidating sociodemographic data, variables, and risk factors associated with HPV infection. The average age of this population was 40 years, ranging from 18 to 78 years (SD: 11.44). The majority self-identified as having a mixed ethnicity, were non-active smokers, and held a high level of education (Table 1).

**Table 1:** Sociodemographic and clinical variables according to the HPV status variables

Sociodemographic characteristics		Total	HPV +	HPV –	p-Value*
			% (n=)	% (n=)	
Age (years)	18–30	95	53 (51)	43 (44)	**0.026**
	31–44	198	40 (79)	60 (119)	
	45–60	132	36 (48)	63 (84)	
	> 60	25	56(14)	44 (11)	
Educational level	Primary	59	32.2 (19)	67.8 (40)	0.263
	Secondary	151	47 (71)	53 (80)	
	Technical	112	41.1 (46)	58.9 (66)	
	Higher	128	43.75 (56)	56.25 (72)	
Ethnicity	White	4	50 (2)	50 (2)	0.536
	Indigenous	1	-	100(1)	
	Mestizo	444	42 (189)	57 (255)	
	Black	1	1 (100)	-	
Active smoker	Yes	4	-	100 (4)	0.083
	No	446	43 (192)	57 (254)	
Gestations	None	58	58.6 (34)	41.4 (24)	0.06
	1 a 2	175	41.8 (73)	58.2 (102)	
	3 a 4	172	61.6 (106)	38.4 (66)	
	> 4	45	42.2 (19)	57.8 (26)	
Sexual partners	1	213	39.4 (84)	60.6 (129)	0.316
	2	113	49.6 (56)	50.4 (57)	
	3	77	44.2 (34)	55.8 (43)	
	> 3	47	38.3 (18)	61.7 (29)	
Menopause	Yes	97	41.2 (40)	58.8 (57)	0.748
	No	353	43.1 (152)	56.9 (201)	
Age of first sexual relationship	<=18	265	40.8 (108)	59.2 (157)	0.326
	> 18	185	45.4 (84)	54.6 (101)	
Protection method	None	169	45.6 (77)	54.4 (92)	0.177
	Barrier	126	35.7 (45)	64.3 (81)	
	Hormonal	155	45.2 (70)	54.8 (85)	
STI	None	408	41.9 (171)	58.1 (237)	0.46
	Viral infection	32	53.1 (17)	46.9 (15)	
	Bacterial infection	10	40 (4)	60 (6)	
Sub-region	Coastal	18	38.9 (7)	61 (11)	0.94
	Lower Sinú	11	45.4 (5)	54.5 (6)	
	Middle Sinú	307	41.7 (128)	58.3 (179)	
	Savannah	12	66.7 (8)	33.3 (4)	
	Upper Sinú	37	48.6 (18)	51.3 (19)	
	San Jorge	36	36.1 (13)	63.9 (23)	
	Sucre	3	66.7 (2)	33.3 (1)	
	Urabá	26	42.3 (11)	57.7 (15)	

*Values in bold < 0,05

* STI: sexually transmitted infection

Analysis of population data showed a notable correlation between the age of women and HPV presence in the group aged 36 to 41 years (Mann-Whitney *p* = 0.035), contrasting with women of different ages with no infection.

Three primer sets were concurrently used for viral genotyping, considering previous studies that showed their ability to enhance the robustness of molecular detection analyses tailored to specific target characteristics [27]. The 42.7% (*n* = 192) of the HPV infections detected were performed by at least one of the three primer sets (Table 2).

**Table 2:** HPV detailed amplification data of each primer set and combination

Primers set	Primers amplification	
	*n*=	%
MY09/11	23	12
GP5+/6+	54	28
pU1R/2 M	41	21
MY09/11 - GP5+/6+	15	8
MY09/11 - pU1R/2 M	9	5
GP5+/6+ - pU1R/2 M	28	15
MY09/11 - GP5+/6+ - pU1R/2 M	22	11

The GP5+/6 + set showed higher identification of HPV DNA in the analyzed population, with 62% of the 192 positive samples. As a result, the overall prevalence was 26% (*n* = 119). In the second place, the HPV detection made by high-risk primer set (pU1R/2 M) represented 52% positivity, revealing 22% (*n* = 100) of the overall population, while MY09/11, with 69 samples detected, performed 36% of the samples (15% of the overall population).

**Fig. 1:**
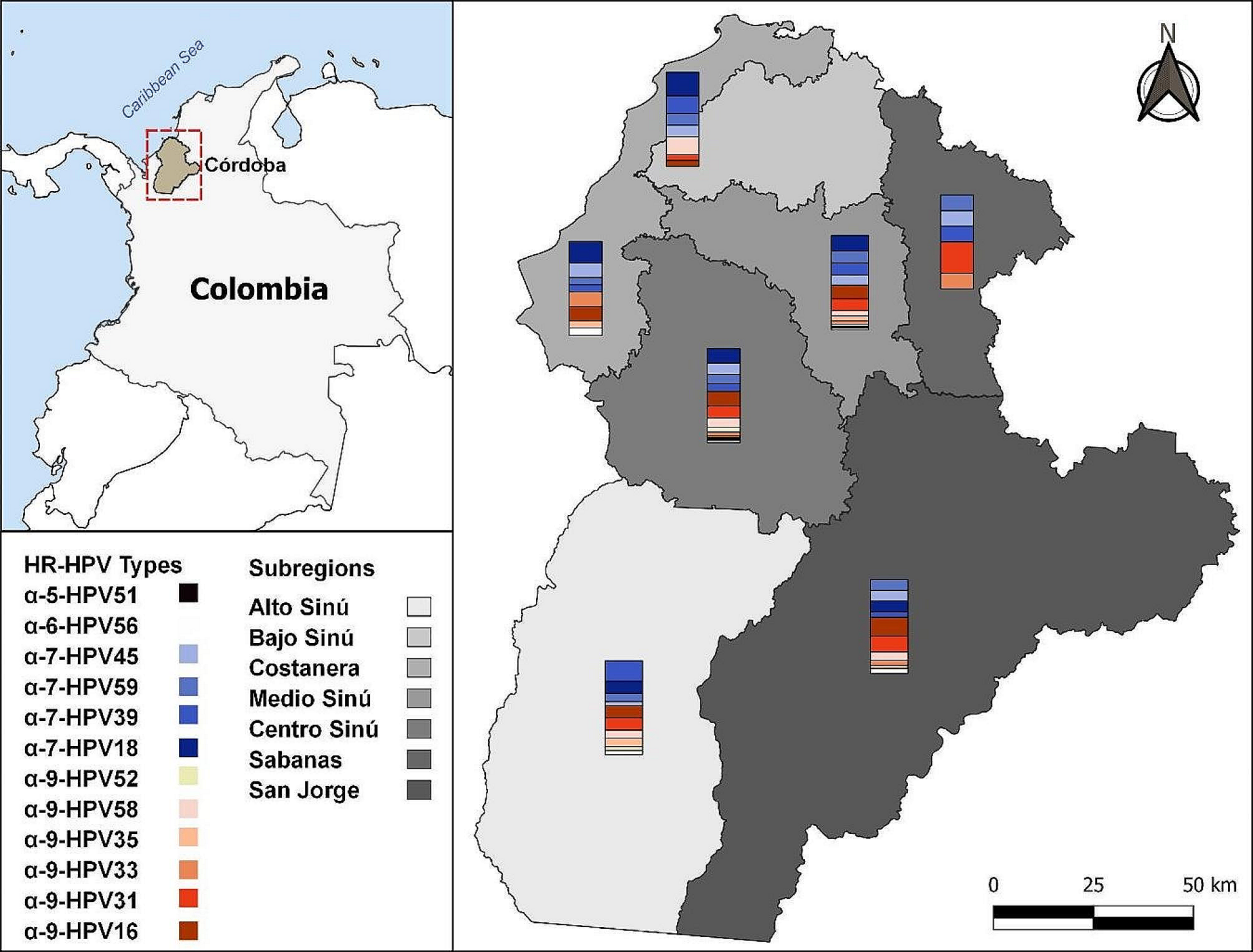
Type-specific frequency distribution of hr-hpv in cordoba department’s subregions

During the assessment of positive samples, the observed viral frequencies were as follows: HPV-18 (13.6%), HPV-16 (12.9%), HPV-31 (11.3%), HPV-45 (10%), HPV-39 (9.3%), HPV-59 (8.7%), HPV-58 (7.3%), HPV-33 (3.8%), HPV-52 (3.6%), HPV-35 (2.7%), HPV-51 (2.4%), and HPV-56 (1.6%). These findings were depicted graphically to illustrate the distribution across various subregions in the Cordoba department (Fig. 1). Notably, a higher prevalence of viral types classified under the Alphapapillomavirus 7 (α7) species was identified within the populations residing in the central and northern subregions of the department [28].

**Fig. 2:**
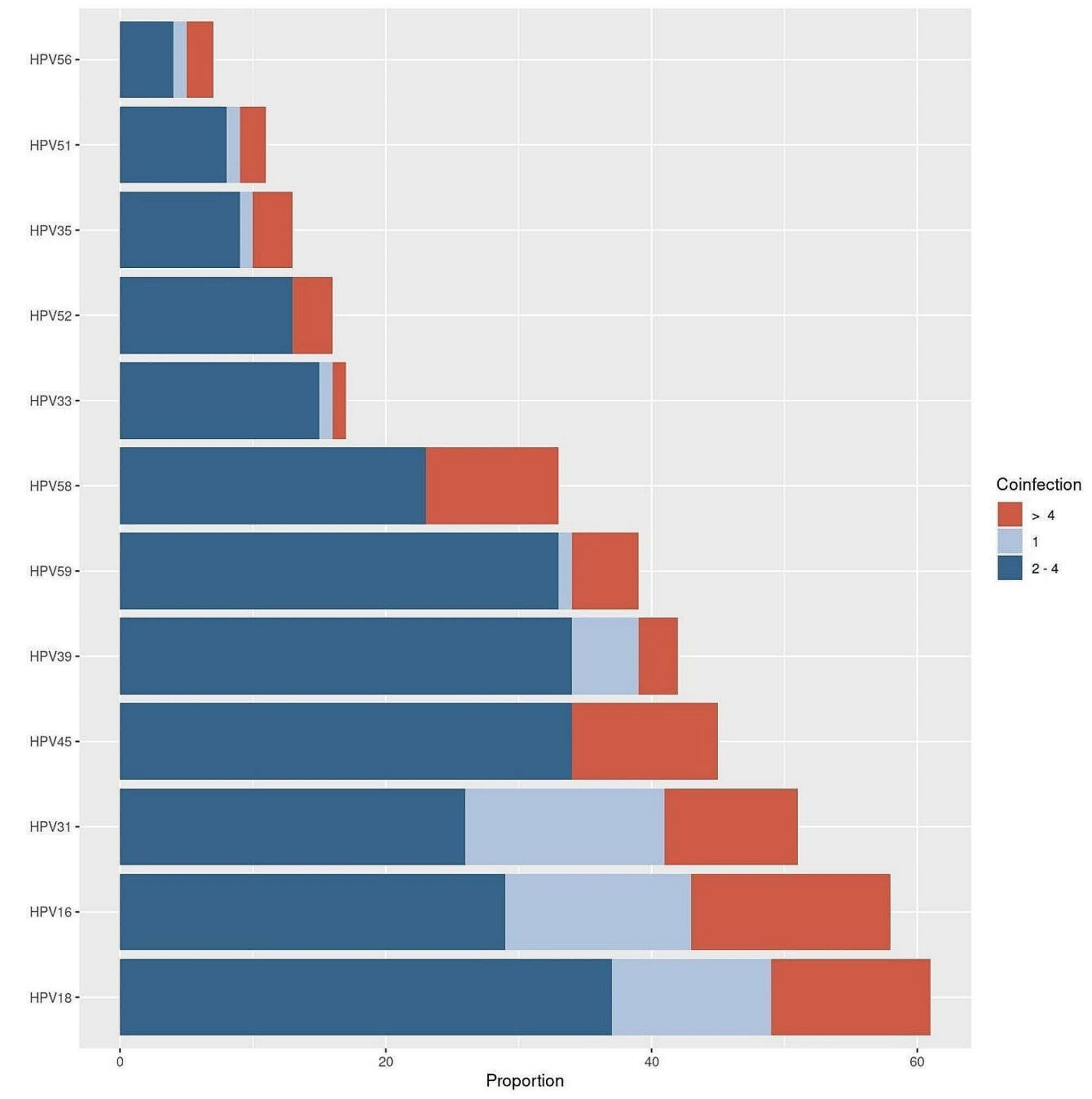
HPV prevalence and infection status distribution by specific high-risk types analyzed

HPV infections were distributed in single infections with a prevalence of 26.5% (*n* = 51) being the most prevalent types: HPV-31 (*n* = 15), HPV-16 (*n* = 14), and HPV-18 (*n* = 12), and multiple infections with a 76.4% (*n* = 141) of prevalence with HPV-18 (*n* = 49), HPV-45 (*n* = 45), HPV-16 (*n* = 44) leading the frequencies (Table 3). Multiple infections were categorized into two groups: infections involving 2 to 4 viral types and those with five or more viral types simultaneously. It is important to notice that HPV-45, -52, and − 58 were only present in cases of coinfection (Fig. 2).

**Table 3:** HPV distribution patterns by infection status

Viral type	HPV status*						Total
	Single		2 to 4 types		> 4types		
	*n*=	%	*n*=	%	*n*=	%	
HPV-16	14	24,14%	27	46,55%	17	29,31%	58
HPV-18	12	19,67%	34	55,74%	15	24,59%	61
HPV-31	15	29,41%	25	49,02%	11	21,57%	51
HPV-33	1	5,88%	15	88,24%	1	5,88%	17
HPV-35	-	-	9	75,00%	3	25,00%	12
HPV-39	5	11,90%	32	76,19%	5	11,90%	42
HPV-45	-	-	33	73,33%	12	26,67%	45
HPV-51	1	9,09%	8	72,73%	2	18,18%	11
HPV-52	-	-	12	75,00%	4	25,00%	16
HPV-56	1	14,29%	4	57,14%	2	28,57%	7
HPV-58	-	-	20	60,61%	13	39,39%	33
HPV-59	1	2,56%	30	76,92%	8	20,51%	39

*Percentages are calculated on the total number of type-specific detections reported

When considering the frequency of each HPV type based on infection status, the most common multiple infections involved the simultaneous detection of 2 to 4 viral types. In this category, HPV-18 was the most frequently identified (*n* = 34), followed by HPV-45 (*n* = 33) and HPV-39 (*n* = 32), all classified under the α7 species. In instances where 5 or more viral types were present, HPV-16 was predominant, detected in 22% of cases (*n* = 17), followed by HPV-18 (20%; *n* = 15), HPV-58 (17%, *n* = 13), HPV-45 (16%; *n* = 12), and HPV-31 (14%; *n* = 11). Other viral types showed frequencies below 10%.

An ordinal logistic regression analysis was conducted to identify factors associated with infection status (Table 4). The analysis revealed that women aged over 60 were more prone to exhibit a higher number of simultaneously infecting HPV types compared to those under 30 (OR = 6.1, 95% CI 1.18–31.63). However, when categorizing women based on menopausal status, it emerged that those who had already experienced menopause showed a protective effect against multiple HPV-type infections (OR = 0.31; 95% CI 0.11–0.89). Notably, participating women reported experiencing menopause from the age of 35 onwards, and 19.6% (*n* = 19) were under 50 years old.

**Table 4:** Ordinal regression with the infection status as the dependent variable

Variable	Categories	OR	z	*P*>|z|	[95% CI]
STI**	None	*-*			
	Viral	2.54	1.73	0.08	[0.88–7.31]
	Bacterial	2.70	0.95	0.34	[0.35–20.99]
Contraceptive method	None	*-*			
	Barrier	0.79	-0.60	0.55	[0.37–1.7]
	Hormonal	1.01	0.02	0.98	[0.52–1.96]
Age	18–30	*-*			
	31–44	1.49	1.10	0.27	[0.73–3.06]
	45–60	2.29	1.55	0.12	[0.8–6.53]
	**> 60**	**6.10**	**2.16**	**0.03**	**[1.18–31.63]**
Age of first sexual relationship		0.72	-0.98	0.33	[0.38–1.38]
Lifetime sexual partners	1	*-*			
	2	1.52	1.24	0.21	[0.78–2.94]
	3	0.75	-0.71	0.48	[0.33–1.67]
	> 3	1,31	0.54	0.59	[0.48–3.6]
**Menopause**		**0.31**	**-2.17**	**0.03**	**[0.11–0.89]**
Births		0.92	-0.79	0.43	[0.76–1.12]

*Values in bold for *p*<0.05

** STI: sexually transmitted infection

## Discussion

The cohort of women examined represents adults who regularly participate in cervical cancer prevention screenings, showing a commendable commitment to their health. The 42% overall prevalence of HPV infection aligns with similar findings in other regions of the country that utilize a strategy involving multiple primer sets for detecting the virus [17, 29, 30].

The sets used in this study are directed toward the identification of the L1 region (MY09/MY11 and GP5+/GP6 + primer sets) and early viral genes (E6–E7) (pU1M/2R), detecting a wide range of HPV types [31, 32]. This study indicated that the GP5+/GP6 + set performed the greatest HPV DNA recognition, followed by high-risk-directed primers. It is understood that the GP + set allows the amplification of single HPV infections and low-copy samples, and pU1M/2R favors identifying abnormal cytological findings [33]. Nevertheless, this work presented high coinfection rates, and these primer set combinations could identify them, which supports the use of multiple primer sets to strengthen HPV molecular epidemiology studies.

Typing the HPV holds significant clinical relevance, given its pivotal role in patient care and treatment planning. This process proves valuable in crafting programs aimed at preventing and treating CC, alongside its role in epidemiological tracking and evaluating vaccination effectiveness within specific regions. In the Caribbean region of Colombia, a higher prevalence of infections from alphapapillomavirus 7 (α7) types was observed, with HPV-18 slightly leading the pack. Interestingly, in other areas like the Amazonas department, Cali, and other central cities, HPV-18 ranked third or fourth in prevalence [17, 29, 30].

The significance of α7 family HPV types lies in their association with severe lesions and adenocarcinomas. Identifying this latter cancer type through cervical cytology tests is often challenging, typically leading to diagnosis in later stages [34, 35]. Moreover, in terms of specific viral dynamics, studies in Colombia have demonstrated that HPV-18 persists for over six months in women undergoing monitoring, surpassing the persistence of other HPV types, sometimes lasting for several years [36]. In fact, viral persistence has been associated with the development of cervical lesions [37].

Colombia has implemented a national HPV vaccination program to protect against HPV-16 and − 18, the most frequent HR-HPV types worldwide. The vaccination scheme in Colombia aims to provide protection against these high-risk HPV types and reduce the risk of developing cervical cancer [38].

The HPV vaccination program initially saw high acceptance, with over 96% vaccination rates in 2012 and 2013. However, safety concerns in 2014 triggered a significant decline, resulting in a low of 14% for initial doses by 2016. Efforts to improve public trust and understanding of the program have since facilitated a rebound, with first-dose rates rising to 39.4% by 2021. Vaccine hesitancy has been linked to insufficient awareness, safety doubts, and connections made between HPV and sexuality [39].

It is necessary to recognize that while HPV-16 and − 18 are the predominant types associated with cervical cancer, the rising prevalence of other high-risk HPV types also demands attention, as has been reported in this work with HPV-45 and − 31 and in other Colombian regions [17]. These types need to be included in enhanced vaccination strategies to reduce further the morbidity and mortality from cervical cancer in Colombia, especially since newer vaccines, with a broader range of HPV types, are becoming available, offering more comprehensive protection against a broader spectrum of HPV infections [40].

One of the most interesting findings involves the association between being over 60 years old and the heightened incidence of infecting viral types. This association gains importance due to the substantial population size within this age group. This implies that viral reactivations, along with sexual behavior, might hold significance within this particular demographic. In Colombia, the viral screening protocol aligns with women’s age groups and their high-risk HPV residence considerations, akin to cervical cytology. It follows a 1-5-5 schedule (every 5 years) for negative results [29]. Considering the insights from this study, this population should also undergo viral monitoring to identify potential viral reactivations.

The increase in multiple HPV infections among older women, even in those with few sexual partners, is believed to be caused by viral reactivation during adulthood. Nonetheless, certain studies propose that changes in sexual behavior among women aged 50 to 60 might also occur due to a rising rate of divorces within this age [41]. However, limitations of the study include the lack of in-depth information regarding the sexual behaviors of the participants. We only considered the count of sexual partners, and no statistically significant connections were established. However, further exploration of this kind of information holds relevance, considering these women spent over 40 years without access to HPV molecular identification techniques during a no-vaccination period against the virus. This emphasizes the need to examine viral dynamics during latent HPV infections in older adult women.

Today, cervical cancer shows elevated incidence rates among women aged 35 to 40 and between 65 and 80 years old. In Colombia, there’s an observed secondary peak in incidence around 55 [42, 43]. However, it has been documented that after menopause, there is a population group where HPV infections are even more frequent, surpassing the frequency observed in other age groups. This rise in incidence might be due to factors such as aging, ovarian function loss, changes in vaginal mucosa, and reduced levels of lactobacilli. Furthermore, it’s established that the composition of vaginal microecology is associated with HPV infection status [44].

Our results showed that women experiencing menopause had a lower number of HPV viral types compared to those who were not in this stage, suggesting a low risk against simultaneous HPV-type infections. Notably, approximately 20% of the menopausal women in this study were under 50 years old. Many studies worldwide have explored the presence of HPV in menopausal women, considering immunological characteristics, age, and factors related to hormonal changes and microbial imbalances. Estrogen has been found to reduce susceptibility to initial HPV infection. However, in cases of persistent HPV infection, sex steroid hormones like estrogen and progesterone may facilitate viral persistence and the progression to cervical cancer [45, 46].

Observations in postmenopausal women reveal that HPV typing offers crucial clinical insights that supplement cervical cytology. This is notable because, at times, normal cytology results showing positive HR-HPV are mistakenly labeled as ASCUS (Atypical Squamous Cells of Undetermined Significance). Yet, upon closer examination through detailed colposcopic analyses, notable percentages of other lesions, particularly high-prevalence cervical dysplasias, have been detected in these ages [47].

In this scenario, alterations occurring in the post-menopause uterus (typically around 47 years in some instances) might reduce the simultaneous presence of various HPV types, promoting competition among them to colonize cervical tissue solely with one HR-HPV type. This occurrence has been linked to the development of lesions and instances of cancer, as studies attribute cancer onset to the tissue colonization effects caused by a singular viral type [35, 48]. Moreover, advancing age might trigger the reactivation of different latent viral types, potentially explaining the identification of multiple infections among populations over 60, as indicated in this study.

A limitation of the study includes the lack of colposcopic information and biopsies from patients because these couldn’t be performed on all women who tested positive for HPV. Including this clinical information would be necessary for future studies.

Finally, when examining cervical cytology results, it becomes clear that there’s a need to identify and characterize the microorganisms found in the samples and comprehend the infection patterns within this population. This is crucial because samples displaying cellular abnormalities also showed concurrent infections involving bacteria or changes in cervical microbiota. Moreover, tracking these women using viral detection tests would be beneficial, possibly incorporating self-sampling methods for monitoring within our older adult communities. This study establishes a fundamental overview of HPV infections in the region and lays the groundwork for potentially integrating the nonavalent vaccine into local vaccination programs in the future. No cost-effectiveness studies have assessed its viability in this population [49].

## Conclusions

This study plays a crucial role in providing baseline information. Considering reports of variations based on the analyzed geographic region, this study allows for identifying and typing HPV in the Colombian Caribbean region, which lacked previous data. The results revealed that the most frequent viral types in the Central and Middle Sinú subregions belonged to the α7 HPV species. An important finding of the study was a strong association observed between women over 60 years old and the presence of simultaneous infections with a higher number of high-risk HPV types. However, among menopausal women, a decrease in infectious high-risk HPV types was observed.

## Data Availability

The datasets used and analyzed during the current study are available from the corresponding author upon reasonable request.
